# Differential Activity of *Striga hermonthica* Seed Germination Stimulants and *Gigaspora rosea* Hyphal Branching Factors in Rice and Their Contribution to Underground Communication

**DOI:** 10.1371/journal.pone.0104201

**Published:** 2014-08-15

**Authors:** Catarina Cardoso, Tatsiana Charnikhova, Muhammad Jamil, Pierre-Marc Delaux, Francel Verstappen, Maryam Amini, Dominique Lauressergues, Carolien Ruyter-Spira, Harro Bouwmeester

**Affiliations:** 1 Laboratory of Plant Physiology, Wageningen University, Wageningen, the Netherlands; 2 Laboratoire de Recherche en Sciences Végétales, Unité Mixte de Recherche (UMR) 5546, Université de Toulouse, Castanet-Tolosan, France; 3 Laboratoire de Recherche en Sciences Végétales, Unité Mixte de Recherche (UMR) 5546, Centre National de la Recherche Scientifique (CNRS), Castanet-Tolosan, France; 4 Centre for Biosystems Genomics, Wageningen, the Netherlands; 5 Bioscience, Plant Research International, Wageningen, the Netherlands; University of Tartu, Estonia

## Abstract

Strigolactones (SLs) trigger germination of parasitic plant seeds and hyphal branching of symbiotic arbuscular mycorrhizal (AM) fungi. There is extensive structural variation in SLs and plants usually produce blends of different SLs. The structural variation among natural SLs has been shown to impact their biological activity as hyphal branching and parasitic plant seed germination stimulants. In this study, rice root exudates were fractioned by HPLC. The resulting fractions were analyzed by MRM-LC-MS to investigate the presence of SLs and tested using bioassays to assess their *Striga hermonthica* seed germination and *Gigaspora rosea* hyphal branching stimulatory activities. A substantial number of active fractions were revealed often with very different effect on seed germination and hyphal branching. Fractions containing (−)−orobanchol and *ent*-2'*-epi-*5-deoxystrigol contributed little to the induction of *S. hermonthica* seed germination but strongly stimulated AM fungal hyphal branching. Three SLs in one fraction, putative methoxy-5-deoxystrigol isomers, had moderate seed germination and hyphal branching inducing activity. Two fractions contained strong germination stimulants but displayed only modest hyphal branching activity. We provide evidence that these stimulants are likely SLs although no SL-representative masses could be detected using MRM-LC-MS. Our results show that seed germination and hyphal branching are induced to very different extents by the various SLs (or other stimulants) present in rice root exudates. We propose that the development of rice varieties with different SL composition is a promising strategy to reduce parasitic plant infestation while maintaining symbiosis with AM fungi.

## Introduction

Parasitic plants of the genus *Striga* are economically important species that parasitize the dicotyledonous cowpea, and cereal crops such as rice sorghum and maize [1]. In the most affected areas, parasitic plants constitute a major constraint to food production and efficient control methods are scant. *Striga* seeds will only germinate after exposure to host derived molecules, called germination stimulants that the parasite uses to detect host presence. The first phases of root parasitism occur underground and the presence of the parasite is difficult to diagnose until the emergence of its shoots. However, crop yield is already compromised at that stage making timely control of this pest even more difficult [2], [3]. It is therefore important to develop control strategies that act before infection is initiated, for example by avoiding or reducing germination of the parasites' seeds. Strigolactones (SLs) are the best described class of germination stimulants and a reduction in the production of these compounds indeed resulted in reduced *Striga* infection [4]–[6]. However, SLs are also signaling compounds for the establishment of symbiosis with arbuscular mycorrhizal (AM) fungi and are plant hormones that modulate plant architecture [7]–[12] and therefore, non-discriminate reduction of their production would likely have negative side effects. The symbiotic AM fungi perceive SLs and respond with extensive pre-symbiotic hyphal branching, thus increasing the efficiency of root colonization. In this symbiotic interaction, the fungus takes up nutrients (especially phosphate and nitrogen) and water from the soil and supplies them to the plant in exchange for carbon assimilates [13]. Plants under phosphate starvation increase production and release of SLs into the rhizosphere to promote the symbiosis [14]–[16]. In soils contaminated with seeds of the parasitic plants, low phosphate availability results in increased levels of infestation by parasitic plants [4].

Adaptation responses to low phosphate such as reduced shoot branching and root system expansion are mediated by SLs [11], [17], [18]. SL biosynthetic mutants suffer, to some extent, from reduced symbiosis with AM fungi and exhibit altered plant shoot and root architecture which may negatively affect crop yields [9]–[11], [19], [20].

**Figure 1:**
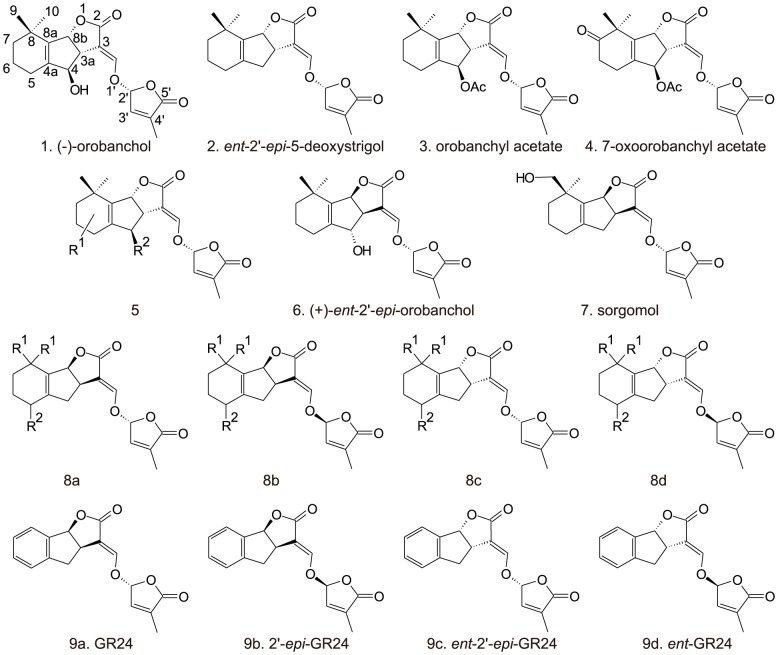
SL structures present in rice root exudates or tested in a seed germination or hyphal branching bioassay. (**1**) (−)−orobanchol; (**2**) *ent*-2'*-epi-*5-deoxystrigol; (**3**) orobanchyl acetate and (**4**) proposed structure of 7-oxoorobanchyl acetate [23]; (**5**) proposed structure of methoxy-5-deoxystrigol isomers (R^1^ = OMe; R^2^ = H) or the methyl ether of orobanchol (R^1^ = H; R^2^ = OMe); (**6**) (+)-*ent*-2'-*epi*-orobanchol; (**7**) sorgomol; (**8a**–**d**) stereoisomers of strigol (R^1^ = CH_3_; R^2^ = OH); 5-deoxystrigol (R^1^ = CH_3_; R^2^ = H), stereochemical configurations **8a** and **8c** are natural (strigol-type and orobanchol-type, respectively) while configurations **8b** and **8d** are not naturally occurring; (**9a**–**d**) stereoisomers of SL analogue GR24.

SLs are derived from all-*trans*-β-carotene that is isomerized into 9-*cis*-β-carotene by β-carotene isomerase D27 (DWARF27) followed by two consecutive cleavage steps by CAROTENOID CLEAVAGE DIOXYGENASE 7 (CCD7; HIGH TILLERING DWARF1 - HTD1/DWARF17 – 17 in rice) and CAROTENOID CLEAVAGE DIOXYGENASE 8 (CCD8; DWARF10 – D10 in rice) resulting in the production of carlactone [21]. The biosynthetic steps that convert carlactone to SL are not yet elucidated. SLs are a reasonably large class of natural compounds consisting of over 15 structural variants, most of which differ only by having one instead of two methyl groups on the cyclohexenyl A-ring or by having various combinations of hydroxyl or acetoxyl substituents on the A- and B-rings [22]. SLs occur in two distinct stereochemical configurations and the stereochemistry of some SLs was recently revised [23]. SLs from the orobanchol-like family have an *ent* oriented C-ring (Figure 1, structures **1**–**5** and **8c**). In the strigol-like family the C-ring has the opposite chirality of the orobanchol-like family (Figure 1, structures **6**;**7** and **8a**) [23]. Plants produce a mixture of SLs that differs between and sometimes even within species [16], [24], [25]. So far, only orobanchol-like SLs have been identified in rice: (−)−orobanchol (**1**), *ent*-2'*-epi-*5-deoxystrigol (**2**), orobanchyl acetate (**3**), 7-oxoorobanchyl acetate (**4**) [10], [23]. In addition, three putative methoxy-5-deoxystrigol isomers (**5**) have been reported with unknown structure and stereochemistry [4].

Parasitic plant seeds and AM fungi have different sensitivities to different SL variants [26], [27]. Interestingly, it was reported that orobanchol-like SLs (of the same type as found in rice exudates) are considerably less active at inducing *Striga hermonthica* seed germination [27]. Here, we extensively survey the chemical composition (SL content) and biological activity of rice root exudates to understand the relevance of the different SLs, and possible other signalling molecules, in the establishment of mycorrhizal symbiosis with the AM fungus, *Gigaspora rosea*, and infection by the parasitic plant, *Striga hermonthica*.

## Materials and Methods

### Strigolactone standards

The synthetic SL GR24 (**9a**–**d**) and (±)-strigol (**8a,d**, R^1^ = CH_3_; R^2^ = OH) were kindly provided by Prof. Binne Zwanenburg (Radboud University Nijmegen, Netherlands); (−)−orobanchol (**1**) and (+)-*ent*-2'-*epi*-orobanchol (**6**), solanacol, orobanchyl acetate (**3**), 7-oxoorobanchyl acetate (**4**), 7-oxoorobanchol, and sorgomol (**7**) were provided by Prof. Koichi Yoneyama (Utsunomiya University, Japan); (±)-2'-*epi*-strigol (**8b,c**, R^1^ = CH_3_; R^2^ = OH), (±)-2'-*epi*-5-deoxystrigol (**8b,c**, R^1^ =  CH_3_; R^2^ = H) and (±)-5-deoxystrigol (**8a,d**, R^1^ = CH_3_; R^2^ = H) were a gift from Prof. Tadao Asami (University of Tokyo, Japan) (Structures **1** to **8a**–**d** represented in Figure 1).

### Plant growth and root exudate collection

The exudates were collected from rice seedlings of the variety Nipponbare and the SL biosynthetic mutant line *d10-2* with Nipponbare background, kindly provided by Prof. Junko Kyozuka (University of Tokyo, Japan) [10]. The seeds were sown in pots of 14 cm diameter filled with quartz sand. The experiment was conducted with three pots per treatment. One pot containing 25 plants represents one replicate. Plants were watered every three days during the first week and every two days during the remaining weeks to full substrate saturation with half-strength modified Hoagland nutrient solution containing NH_4_NO_3_ (5.6 mM), K_2_HPO_4_ (0.4 mM), MgSO_4_ (0.8 mM), FeSO_4_ (0.18 mM), CaCl_2_ (1.6 mM), K_2_SO_4_ (0.8 mM), MnCl_2_ (0.0045 mM), CuSO_4_ (0.0003 mM), ZnCl_2_ (0.0015 mM), Na_2_MoO_4_ (0.0001 mM). After 3 weeks, phosphate starvation and phosphate starvation in combination with 0.01 µM fluridone – an inhibitor of carotenoid and therefore SL biosynthesis – were applied. Control plants were watered with the half-strength modified Hoagland nutrient solution described above. For the phosphate starvation treatment, KNO_3_ (0.8 mM) was substituted for K_2_HPO_4_ to maintain the same the K^+^ concentration. Residual phosphate was removed from the pots by applying 1 L of the concentration nutrient solution and draining the pots. Six days after the start of the treatments the treatment was repeated. Root exudates were collected 24 hours later by applying 1 L of the corresponding nutrient solution and collecting the flow through.

### Sample preparation

The root exudates were concentrated using an SPE cartridge (GracePure™ SPE C18 – Max 500 mg) and eluted in 4 mL of 100% acetone. For HPLC, 250 µL of water was added to 1 mL of this acetone eluent after which the acetone was evaporated under a flow of N_2_. The remaining 250 µL sample was injected into the HPLC and 1 min fractions (corresponding to 1 mL) were collected. The fractions were evaporated to dryness and dissolved in 200 µL water for further analysis. For MRM-LC-MS analysis, 50 µL of the C18 acetone eluent was diluted 3-fold in water and HPLC fractions were diluted 2-fold in water. For seed germination bioassays, C18 acetone eluents (crude exudates) were diluted 32-fold in water and HPLC fractions 5-fold. For the AM fungal hyphal branching bioassay the HPLC fractions of each replicate were pooled and tested in the same concentration as in the seed germination bioassay.

### Fractionation of root exudates

Root exudates were fractioned by HPLC. The samples were injected into a XBridgeTM C18 column (4,6*150 mm from 5 µm, Waters) using a U6K injector (Waters). For the gradient model 510 pumps (Waters) were used. The mobile phase was water and the following gradient to acetonitrile used: 1 min 100% water, 2 min 27% acetonitrile, 15 min 45% acetonitrile, 24 min 80% acetonitrile and 24.2 min 100% acetonitrile which was maintained for 4 minutes to clean the column. The flow rate was 1 mL min^−1^ and the column temperature 25°C. Fractions of one minute were collected using a Biofrac fraction collector (Biorad).

### MRM-LC-MS analysis

For LC-MS analysis, samples were filtered through mini syringe filters (Minisart SRP4). The retention times, mass transitions and MS/MS spectra of available SL standards such as (+)-*ent*-2'*-epi-*orobanchol (**6**), (−)-orobanchol (**1**), (±)-5-deoxystrigol (**8a,d**, R^1^ = CH_3_; R^2^ = H), (±)-2'*-epi-*5-deoxystrigol (**8b,c**, R^1^ =  CH_3_; R^2^ = H), (±)-sorgolactone, (±)-strigol (**8a,d**, R^1^ = CH_3_; R^2^ = OH), solanacol, orobanchyl acetate (**3**), (±)-7-oxoorobanchol and (±)-7-oxoorobanchyl acetate (**4**) were compared with each sample to quantify SLs using ultra performance liquid chromatography coupled to tandem mass spectrometry (UPLC-MS/MS). Analyses were performed using a Waters Xevo tandem quadrupole (TQ) mass spectrometer equipped with an ESI source. Chromatographic separation was achieved on an Acquity UPLC BEH C18 column (150 × 2.1 mm, 1.7 µm) (Waters) by applying a water/acetonitrile gradient to the column, starting from 5% (v/v) acetonitrile for 2.0 min and rising to 50% (v/v) acetonitrile at 8.0 min, followed by a 1.0 min gradient to 90% (v/v) acetonitrile, which was maintained for 0.1 min before going back to 5% (v/v) acetonitrile using a 0.2 min gradient, prior to the next run. Finally, the column was equilibrated for 2.8 min, using this solvent composition. Operation temperature and flow-rate of the column were 50°C and 0.4 mL min^−1^, respectively. Sample injection volume was 15 µL. The mass spectrometer was operated in positive electrospray ionization (ESI) mode. Cone and desolvation gas flows were set to 50 and 1000 L h^−1^, respectively. The capillary voltage was set at 3.0 kV, the source temperature at 150°C and the desolvation temperature at 650°C. The cone voltage was optimized for each SL standard using the IntelliStart MS Console. Argon was used for fragmentation by MS/MS spectra in the collision cell.

The identification of SLs in rice root exudates and extracts was done using Multiple Reaction Monitoring (MRM) and by comparing retention times and MRM mass transitions with those of the available SL standards mentioned above. MRM transitions were optimized for each standard using the IntelliStart MS Console. The MRM transitions for putative 4-methoxy-5-deoxystrigol isomers were initially set based on the theoretically predicted fragmentation (see Results and Discussion section). MRM-transitions for the predicted putative SLs were incorporated in the MRM-method. The structures of all detected SLs were confirmed by MS/MS fragmentation spectra. Data acquisition and analysis were performed using MassLynx 4.1 software (Waters). Full mass scan and precursor ion scan for *m/z* = 97 were performed to search for unknown SLs in biologically active HPLC-fractions 15 to 19. The LC-MS results of the measurements of (−)-orobanchol (**1**) and *ent-*2'*-epi-*5-deoxystrigol (**2**) (of 3 biological replicates) were compared using ANOVA followed by pair wise comparisons with t-test (LSD values) in Genstat (Genstat for Windows 12^th^ Edition; VSN International).

### S. hermonthica seed germination bioassay

Seeds of *Striga hermonthica* used for the bioassay were kindly provided by Bob Vasey and originating from a sorghum field in Sudan, collected in 1995. The bioassays were performed as described [28]. The samples were tested in three technical replicates (three discs) and 3 biological replicates were tested, one independent bioassay per biological replicate. Given the binomial distribution nature of the measurements the mean values of the seed germination scores (3 replicates per treatment per fraction) were compared using a Chi-square test in Genstat (Genstat for Windows 12^th^ Edition; VSN International).

### AM fungal hyphal branching bioassay

For the AM branching bioassay spores of *Gigaspora rosea* (DAOM 194757) were used. The spores were routinely produced in pots containing leek and collected by wet sieving. They were washed in water/0.05% Tween 20 (v/v), soaked with 2% (w/v) Chloramine T (Sigma) for 10 min, washed again three times in sterile water for 30 s per wash, and stored in an antibiotic solution containing 100 mg L^−1^ gentamycin and 200 mg L^−1^ streptomycin. After 2 days at 4 °C, a second treatment with Chloramine T was carried out under the same conditions. They were then stored in the antibiotic solution at 4°C before use. Branching bioassays were carried out according to Buee *et al*. [29]. Four spores of *Gi. rosea* were germinated (in 2% CO_2_ at 30 °C in dark) on M medium (Becard & Fortin, 1988) supplemented with 10 µM quercetin (Sigma) and solidified with 0.6% Phytagel (Sigma). Seven days after inoculation, each spore produced a single germ tube growing upwards. Two small wells on each half of the Petri dish, near the hyphal tip, were made in the gel with a Pasteur pipette tip and 5 µL of the test solution (SL analogue GR24 or purified fraction) or 10% acetonitrile (control) was injected in each well. After 24 h, hyphal branching was recorded by counting newly formed hyphal tips. Twenty to thirty spores were used for each treatment. Values of each tested fraction were compared with the corresponding control using the Student's t-test in Genstat (Genstat for Windows 12^th^ Edition; VSN International).

## Results

**Figure 2:**
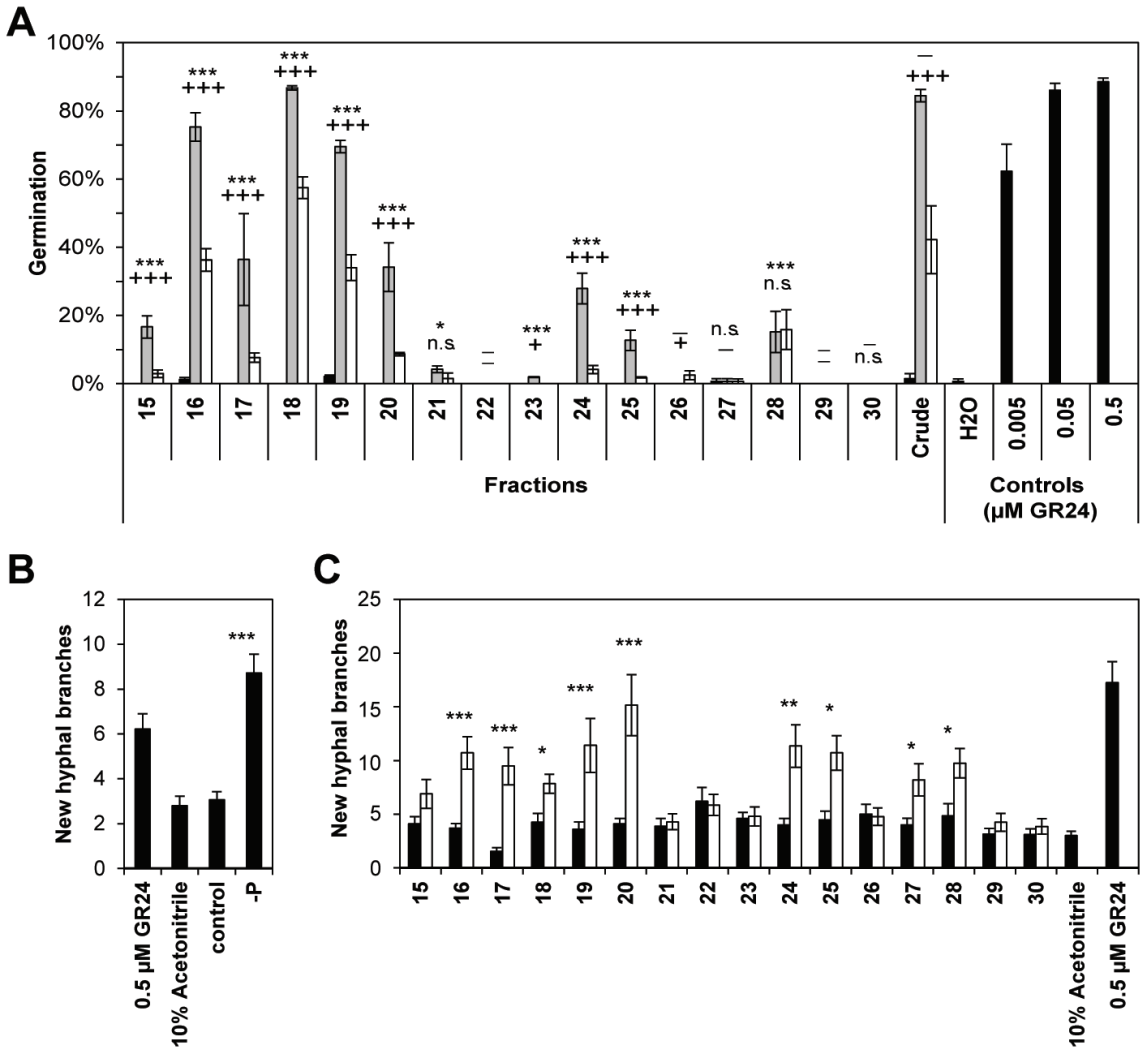
Activity profiles of rice root exudates. Germination of *S. hermonthica* (one biological replicate; the other two are shown in ) obtained with crude exudates and exudate fractions from rice plants (**A**) treated with full nutrition (black bars); phosphate starvation (grey bars) and phosphate starvation plus 0.01 µM fluridone (white bars). Water and SL analogue GR24 (0.005, 0.05 and 0.5 µM) were used as controls. The error bars represent the standard error of 3 technical replicates. Significance levels between treatments as determined using a *X* ^2^ test are indicated: */+  =  *P*<0.05; **/++  =  *P*<0.01; ***/+++  =  *P*<0.001; n.s.  =  *P*>0.05; *  =  control vs. phosphate starvation treatment; +  =  phosphate starvation vs. phosphate starvation plus fluridone treatment. When germination values are close to zero the statistical test cannot be performed, which is indicated with “−”. AM fungal hyphal branching induced by crude exudates (**B**) and exudate fractions (**C**) of rice treated with full nutrition (black bars) and phosphate starvation (white bars) in germinating *Gi. rosea* spores. The assay was performed with pooled samples of three biological replicates. GR24 (0.005, 0.05 and 0.5 µM) and 10% acetonitrile in water were used as controls. The bars represent the mean of the total number of new branches, the error bar the standard error of the mean (n = 20). Significance values comparing means between control treatments and phosphate starvation treatment are indicated above the bars. (*  =  *P<*0.05, **  =  *P<*0.01, ***  =  *P<*0.001).

Rice root exudates were profiled to find compounds responsible for *S. hermonthica* seed germination and AM fungi hyphal branching. Plants were submitted to phosphate starvation with and without the application of 0.01 µM fluridone, an inhibitor of carotenoid and hence SL biosynthesis [28]. A reduction in seed germination stimulatory activity of root exudate fractions by fluridone treatment would suggest that the compound(s) responsible for the biological activity in that fraction is a (are) SL(s). The root exudates were fractioned by HPLC and the biological activity of the resulting fractions as well as the crude exudates (not fractioned) was assessed. Each of the three biological replicates was tested in independent seed germination bioassays of which one is shown in Figure 2 A, and the remaining are shown in Figure S1. Preliminary results showed that fractions eluting before 14 min (fractions 1 to 14) do not exhibit seed germination stimulatory activity (data not shown). Fractioned and crude exudates of control plants with sufficient phosphate showed almost no activity across all fractions. The seed germination stimulatory activity was significantly increased by phosphate starvation in crude exudates and in fractions 15–21, 23–25 and 28 (*P*<0.05 using *Χ* ^2^ test). This was observed consistently in all replicates (Figure 2 A and Figure S1). Fluridone treatment significantly reduced the activity of crude exudates and of fractions 15 to 19 and 24) with 99.9% confidence (*P*<0.001 using *Χ* ^2^ test) in all biological replicates. In fractions 20, 21, and 25 fluridone treatment also reduced the activity but the effect was not consistently significant; in two replicates these fractions showed significance at *P*<0.001 using the *Χ* ^2^ test, but not in the third replicate, likely because the germination stimulatory activity in these fractions was sometimes low. Also in fraction 28 that induces low germination activity the fluridone treatment did not significantly reduce seed germination. The effect of crude and fractioned root exudates on AM fungal hyphal branching was also assessed using *Gi. rosea* spores (Figure 2 B and C). Just as for *S. hermonthica* seed germination, phosphate starvation significantly increased branching stimulatory activity of crude exudates and most fractions (16–20, 24–25 and 27–28, at *P*<0.005, using Student t-test). Crude and fractioned exudates of control, non-phosphate starved plants did not show a significant difference in branching activity compared with the negative control, 10% acetonitrile. All the active fractions (of phosphate starvation treated rice) induced hyphal branching to a similar level. Interestingly, however, fraction 18 - the most active in the *S. hermonthica* seed germination bioassay - induced less AM fungal hyphal branching than the other active fractions (Figure 2 C).

**Figure 3:**
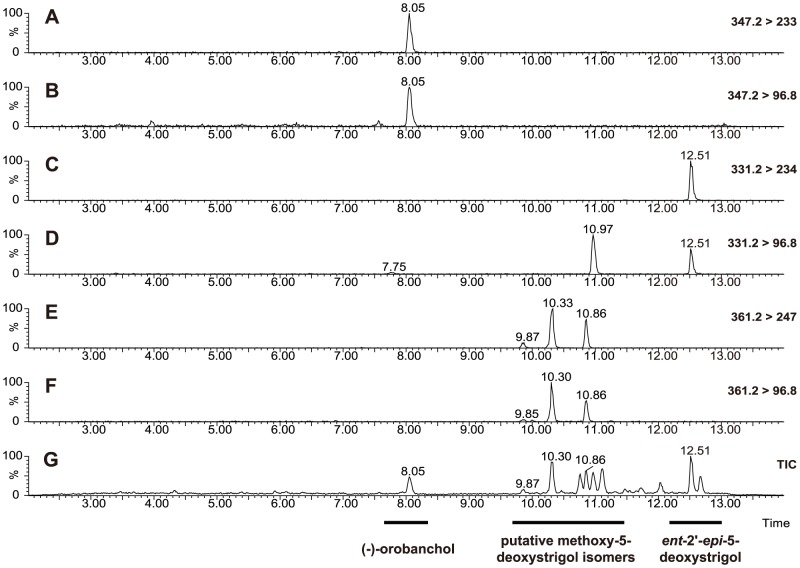
SL analysis of rice root exudates. Root exudates from rice plants grown under phosphate starvation were analyzed with liquid chromatography coupled with tandem mass spectrometry (LC-MS/MS) using multiple reaction monitoring (MRM). Chromatograms of (**A**) transitions 347.2 > 233 and (**B**) 347.2>96.8 for orobanchol; (**C**) transitions 331.2> 234 and (**D**) 331.2>96.8 for 2'*-epi-*5-deoxystrigol; (**E**) transitions 361.2>247 and (**F**) 361.2>96.8 for three putative methoxy-5-deoxystrigol isomers; (**G**) the total ion count (TIC) showing of all measured transitions and where orobanchol (8.05 min), *ent*-2'-*epi*-5-deoxystrigol (12.51 min) and the three putative methoxy-5-deoxystrigol isomers (9.87; 10.33; 10.86 min) are visible.

**Figure 4:**
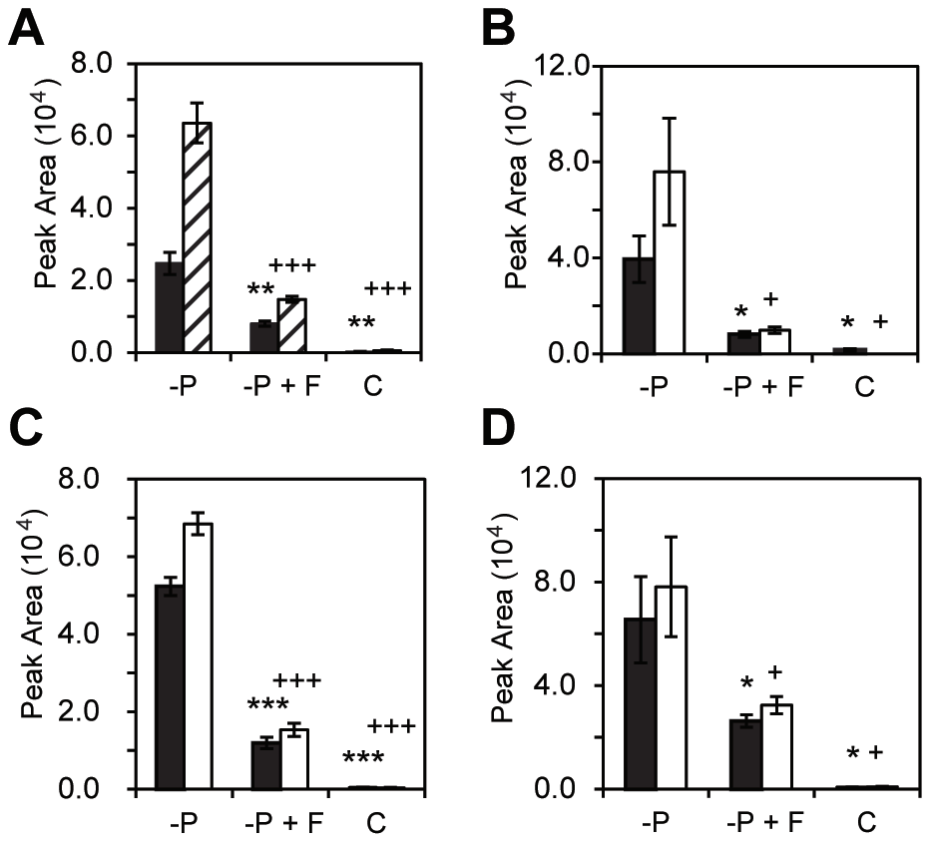
Abundance of (−)−orobanchol, *ent*-2'-*epi*-5-deoxystrigol and putative SL-like compounds in phosphate starvation and fluridone treatments. Peak areas obtained with liquid chromatography coupled with tandem mass spectrometry (LC-MS/MS) analysis using multiple reaction monitoring (MRM) of root exudates of rice. (**A**) (−)−orobanchol (MRM transition 347.2 >96.8; black bars) and *ent*-2'-*epi*-5-deoxystrigol (MRM transition 331.2>234; hatched bars); (**B**–**D**) three putative SL-like compounds measured in crude exudates with the retention times: rt =  9.87 (**B**); rt = 10.3 and (**C**) rt = 10.9 (**D**) and the MRM transitions 361>96.8 (black bars) and 361>247 (white bars). All measurements taken from crude exudates of plants grown in different treatments: phosphate starvation (−P); phosphate starvation combined with fluridone (−P+F) and control treatment with full nutrient supply (C). The error bars represent the standard error of 3 biological replicates. Significance values are indicated with * (for *ent*-2'*-epi-*orobanchol and for 361>96.8 transition) and + (for 2'*-epi-*5-deoxystrigol and for 331.2>234 transition) and compare phosphate starvation (-P) treatment vs. phosphate starvation with fluridone (-P+F) and -P vs. full nutrition (C) (*/+  =  *P<*0.05, **/++  =  *P<*0.01, ***/+++  =  *P<*0.001).

To gain insight into the identity of the compounds responsible for the biological activity, MRM-LC-MS analysis was performed. The MRM chromatograms of crude exudates revealed an intense peak in the channels *m/z* 347>233, 347>205 and 347>97 at retention time 8.05 min, which matches with an authentic standard of (−)-orobanchol (**1**) (Figure 3 A and B). In the channels *m/z* 331>234 and 331>97 there was a peak at 12.51 min, which matches with *ent*-2'-*epi*-5-deoxystrigol (**2**) (Figure 3 C and B). MS/MS fragmentation spectra, and the addition of authentic orobanchol and (±)-2'*-epi-*5-deoxystrigol (**8b,c**, R^1^ =  CH_3_; R^2^ = H) to the samples, confirmed that the compounds detected were indeed orobanchol and *ent*-2'*-epi-*5-deoxystrigol. The same two SLs were barely detectable in exudates of control plants supplied with full nutrient solution, were most abundant in the phosphate starvation treatment and were both significantly reduced by fluridone treatment (*P*<0.001) (Figure 4 A). In addition to (−)−orobanchol (**1**) and *ent*–2'–*epi*–5–deoxystrigol (**2**) three unknown peaks were detected using the channels for 7-oxoorobanchol (*m/z* 361>247 and 361>97) at the retention times 9.9, 10.3 and 10.9 min (Figure 3 E and F), which is substantially later than 7-oxoorobanchol, which elutes at 3.7 min. The three unknown compounds (from here on referred to as methoxy-5-deoxystrigol isomers; see Discussion) were most abundant in the phosphate starvation treatment and were reduced by fluridone treatment (Figure 4 B - D) suggesting they are SL-like compounds.

MRM-LC-MS analysis was also performed on the HPLC fractions to try to correlate the presence of SLs with the seed germination and hyphal branching activity. (−)−orobanchol (**1**) was detected in fractions 19 to 21 with highest abundance in fraction 20 and *ent*-2'-*epi*-5-deoxystrigol (**2**) in fractions 27 and 28 with highest signal in fraction 28 (data not shown). The three methoxy-5-deoxystrigol isomers detected in the channel for 7-oxoorobanchol (*m/z* 361> 247 and 361>97) at the retention times 9.95, 10.3 and 10.9 min eluted in fractions 23-25 with highest abundance in fraction 24 and are likely responsible for the seed germination stimulant/hyphal branching activity peak in fractions 24-25. The activity of the three compounds could not be evaluated individually as they did not separate on HPLC due to their highly similar retention time.

The fractions with highest seed germination inducing activity (16 and 18) were also analyzed using known MRM transitions typical for SLs as well as full mass scan and precursor ion scan for *m/z* = 97. However, we could not detect any masses that could be indicative for SLs and displayed an expected abundance pattern across the treatments similar as the known SLs: low in control, high in P starvation, low upon fluridone treatment.

**Figure 5:**
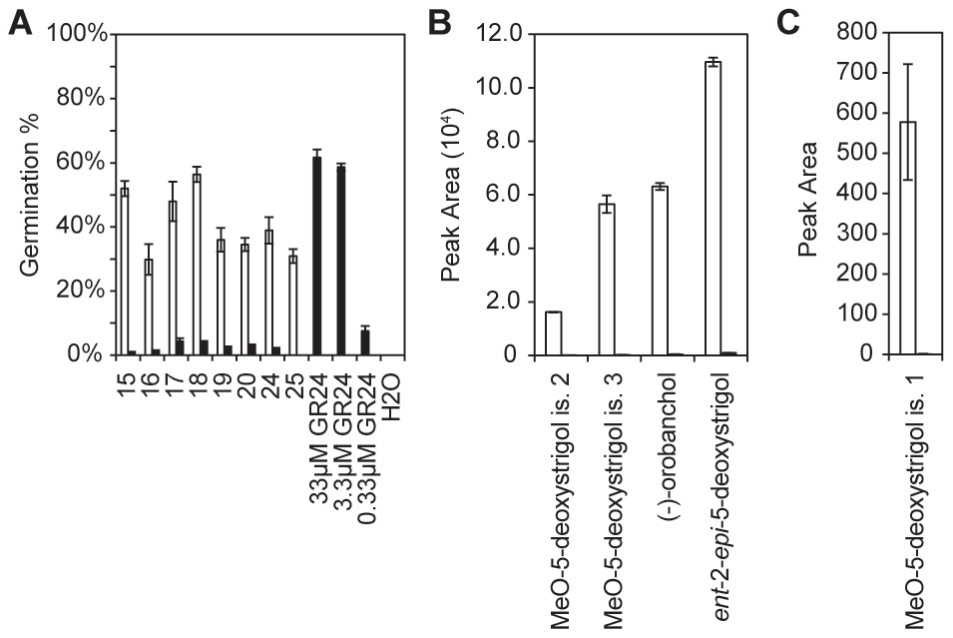
Characterization of rice mutant *d10* exudate. Germination assay with *S. hermonthica* seeds on exudate fractions of full nutrition (black bars) and phosphate starvation (white bars) treated plants (**A**). Water and GR24 (0.33, 3.3 and 33 µM) were used as controls. Peak areas of *ent*-2'-*epi*-5-deoxystrigol, (−)−orobanchol and putative methoxy-5-deoxystrigol isomers 2 and 3 (**B**) and putative methoxy-5-deoxystrigol isomer 1 (**C**) obtained with liquid chromatography coupled with tandem mass spectrometry (LC-MS/MS) analysis using multiple reaction monitoring (MRM) of root exudates of *d10-2* (black bars) and WT (white bars) under phosphate starvation treatment.

To further investigate the nature of the active compounds in fractions 16, 18, 24 and 25, exudates of the SL biosynthetic mutant *d10-2* were studied [10]. HPLC fractions 16–25 collected from *d10-2* and its background Nipponbare were tested using the seed germination bioassay. All active fractions in the wild type had reduced activity in the mutant (Figure 5 A) supporting the SL (CCD8-dependent) nature of the compounds responsible for the biological activity of these fractions.

The MRM-LC-MS spectra of *d10-2* mutant root exudates confirmed that (−)-orobanchol (**1**) and *ent*-2'-*epi*-5-deoxystrigol (**2**) as well as the three methoxy-5-deoxystrigol isomers detected at 9.9, 10.35 and 10.95 were strongly decreased in *d10-2* mutant root exudates (Figure 5 B,C), further indicating that the latter three are SLs/require CCD8. Fractions 16 and 18 of *d10-2* exudates were also analyzed by LC-MS and compared with those from wild type plants using full-scan mass spectrometry, but no differential masses were found that could explain the seed germination activity in the wild type and give a hint on the identity of the seed germination stimulant(s) in these fractions.

The activity profiles obtained with the exudate fractions, when tested with the seed germination and hyphal branching are different. Some fractions that stimulate high seed germination percentages induce low fungal response and the contrary is also observed. To further investigate the differences in activity observed in our bioassays, we performed a seed germination bioassay using pure or racemic mixtures of SLs (Table 1). We observed that sorgomol (**7**) is the most active of the tested SLs inducing 36% seed germination at 200 nM and 26% seed germination at 20 nM followed by (+)-*ent*-2'-epi-orobanchol (**6**, 34% seed germination at 200 nM and 8.7% at 20 nM). The racemates of (±)-strigol (**8a,d**, R^1^ = CH_3_; R^2^ = OH); (±)-5-deoxystrigol (**8a,d**, R^1^ = CH_3_; R^2^ = H) and the racemic mixture of all 4 stereoisomers of GR24 (**9a**–**d**) have intermediate activity inducing 9.3%, 6.0% and 2.0% seed germination at 20 nM and inducing 19%, 26% and 25% seed germination at 200 nM, respectively. The racemate of (±)-2'-*epi*-5-deoxystrigol (**8b,c**, R^1^ =  CH_3_; R^2^ = H) induced less seed germination (11 % at 200 nM) and was not active at 20 nM. The least active SLs were (−)-orobanchol (**1**) and the racemate of (±)-2'-*epi*-strigol (**8b,c**, R^1^ = CH_3_; R^2^ = OH) that induced less than 1% seed germination in both concentrations. Table 1 also summarises data from a study by Akiyama et al. that analysed the *Gigaspora margarita* hyphal branching activity of a range of different SLs [26]. In contrast to what is observed with *S. hermonthica*, both orobanchol (**1**) and *ent*-2'-*epi*-5-deoxystrigol (**2**) are highly active at inducing hyphal branching and their activity is similar to their natural stereoisomers. Strigol (**8a**, R^1^ = CH_3_; R^2^ = OH), sorgomol (**7**), GR24 (**9a**) and (±)-2'-*epi*-strigol (**8b,c**, R^1^ = CH_3_; R^2^ = OH) were considerable less active (100 fold) than orobanchol (**1**), and the remaining GR24 stereoisomers (**9b**–**d**) were 10000- to 1000-fold less active than GR24 (**9a**).

**Table 1:** *Striga hermonthica* germination and *Gigaspora margarita* hyphal branching in the presence of SL standards.

	*S. hermonthica* germination (%)		*Gi. margarita* hyphal branching^1^
	200 nM	20 nM	MEC^2^ in pg per disc
(±)-2'*-epi-*strigol	0.00 ± 0.00	0.00 ± 0.00	100
(−)−orobanchol^3^	0.67 ± 0.67	0.00 ± 0.00	1
*ent*-2'-*epi*-5-deoxystrigol	–	–	3
2'-*epi*-5-deoxystrigol	–	–	30
(±)-2'-*epi*-5-deoxystrigol	11.33 ± 1.76	0.00 ± 0.00	–
GR24	–	–	100
*ent*-GR24	–	–	10000
2'-epi-GR24	–	–	1000
*ent*-2'-epi-GR24	–	–	1000
GR24 (4 stereoisomers)	25.33 ± 1.76	2.00 ± 1.15	
*ent*-5-deoxystrigol	–	–	30
5-deoxystrigol	–	–	3
(±)-5-deoxystrigol	26.00 ± 9.87	6.00 ± 3.05	–
(±)-strigol	19.33 ± 8.82	9.33 ± 4.67	100
*Ent*-2'-*epi*-orobanchol^4^	34.00 ± 1.15	8.67 ± 2.40	1
sorgomol	36.00 ± 3.05	26.00 ± 5.03	100

1 Results extracted from Akiyama et al. [26]; ^2^ MEC  =  minimum effective concentration; ^3^ in Akiyama et al. [26] these compounds are named (+)−orobanchol and (+)-2'-*epi*-orobanchol respectively, before revision of stereochemical structure whereas the present table indicates the revised stereochemistry [23].

## Discussion

Rice root exudates were fractioned to evaluate the contribution of SLs and potentially other signalling molecules to the *S. hermonthica* seed germination stimulant and AMF hyphal branching activity of rice root exudate. MRM–LC–MS analysis of these HPLC fractioned rice root exudates showed the presence of (−)−orobanchol (**1**) in fractions 19, 20 and 21 and *ent*-2'-*epi*-5-deoxystrigol (**2**) in fraction 28 suggesting that these SLs are responsible for the seed germination and hyphal branching stimulatory activities of these fractions. These results confirm the presence of SLs found previously in root exudates of the rice variety Nipponbare except for orobanchyl acetate (**3**) that was not detected in the present study but is reported by others [23]. A fourth SL -7-oxoorobanchyl acetate (**4**) – was also reported to be produced in Nipponbare between days 10 to 17 after germination [23]. In the present study the exudates were collected at a later stage and this SL was not detected.

**Figure 6:**
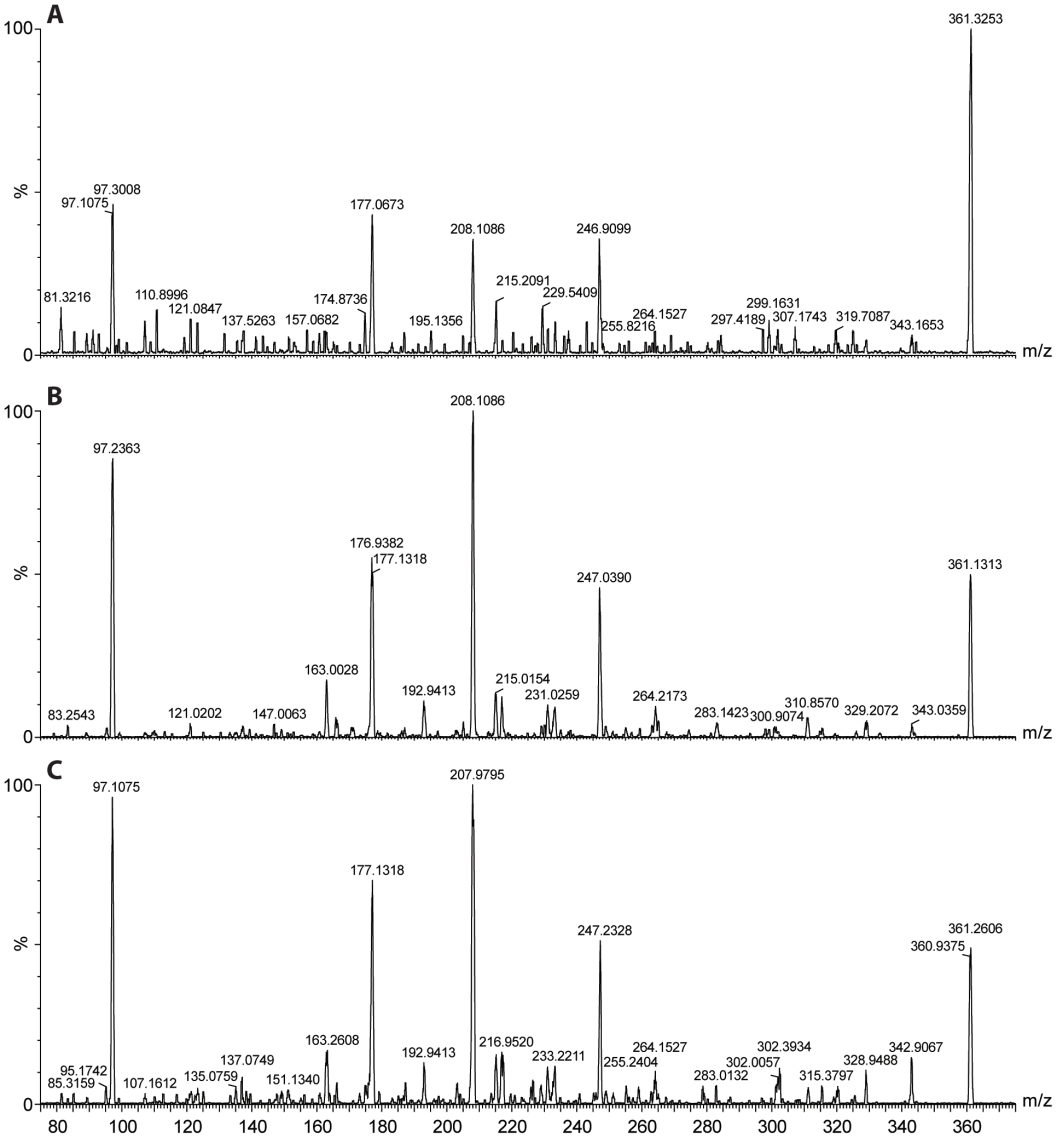
MS/MS spectra of putative SL-like compounds. The spectra were measured at the retention time of each isomer: 9.87 min (**A**), 10.35 min (**B**) and 10.95 min (**C**).

The relative abundance of (−)−orobanchol (**1**) and *ent*-2'-*epi*-5-deoxystrigol (**2**), measured by MRM-LC-MS across the different treatments matches the seed germination stimulatory activity of the fractions where these SLs elute (19–20 and 28 respectively). Phosphate starvation induced the highest production of (−)−orobanchol (**1**) and *ent*-2'-*epi*-5-deoxystrigol (**2**) which resulted in the highest germination of *Striga* seeds. Fluridone application inhibited the biosynthesis of these SLs which resulted in a lower biological activity of the fractions and crude exudates, confirming the inhibitory effect of fluridone on SL production that was previously described [28]. MRM-LC-MS analysis of fractions 24–25 revealed the presence of three compounds with the same mass *m/z* 361 showing up in the 361.2>247 and 361.2>96.8 MRM channels. These metabolites were most abundant in exudates of phosphate starved plants and were reduced by fluridone application. The seed germination activity obtained with fractions 24 and 25 correlates with the abundance of the detected masses. MS/MS analysis of the compounds eluting in fractions 24 and 25 shows fragmentation patterns typical for SLs [30]: loss of the D-ring and H_2_O yields fragment ions [M+H – D-ring - H_2_O]^+^ with *m/z*  =  247 and the fragment ion of the D-ring itself C_5_H_5_O_2_ with *m/z*  =  97 (Figure 6). The loss of methanol [M+H –MeOH]^+^, yielding the fragment ion *m/z*  =  329, is not typical for the fragmentation of known SLs and could indicate the presence of a methoxy-group in the molecule (Figure 6). This feature could explain the late retention time of these putative SLs compared with orobanchol (Figure 3 G) and other known SLs given that methyl ethers are less polar than alcohols (Figure 3 E and G). The MS/MS fragmentation spectra of all three compounds are very similar (Figure 6). Based on these data we suggest that the compounds eluting at 9.5, 10.3 and 10.9 are methoxy-5-deoxystrigol isomers (**5**). Isolation followed by NMR or chemical synthesis should give the final proof of the structure of these three isomers. As we do not have this proof as yet, we will refer to these new compounds under the combined name methoxy-5-deoxystrigol isomers [4]. The absence of the putative methoxy-5-deoxystrigol isomers in *d10-2* exudate further supports that these compounds are produced from the SL pathway (Figure 5 B and C).

Fractions 16 and 18 induced the highest level of *S. hermonthica* germination (Figure 2 and Figure S1) and do not contain any of the SLs discussed above. As mentioned above, two other SLs, orobanchyl acetate (**3**) and 7-oxoorobanchyl acetate (**4**), were recently reported in rice [23]. Orobanchyl acetate (**3**) elutes after (−)-orobanchol (**1**) and is unlikely to be responsible for the activity in fractions 16 and 18. 7-Oxoorobanchyl acetate (**4**) elutes before (−)−orobanchol (**1**) and could be present in fraction 16 to 18. This SL was previously detected in exudates collected 10 to 17 days after germination [23]. In the present study, exudates were collected in later stages and this SL was not detected in crude exudates nor in any of the fractions. We could also not detect any other known SLs in fractions 16 to 18; however the seed germination bioassays showed that the activity of fractions 16 and 18 followed the same trend across the treatments as the activity of SL containing fractions. They were increased by phosphate starvation and reduced by fluridone application (Figure 2 A). The seed germination stimulatory activity of these fractions in *d10-2* root exudate was also clearly reduced (Figure 5 A). All this strongly suggests that the activity in these fractions is caused by compounds derived from the SL pathway after carlactone (as CCD8 is required for their production). Considering their high activity in the induction of *S. hermonthica* germination it is of great interest to identify these compounds.

Overall, the activity profiles for *S. hermonthica* seed germination and AM fungal hyphal branching are similar but not the same (Figure 2). All active fractions in the seed germination bioassay exhibited hyphal branching stimulatory activity albeit to a different extent. Fraction 20 [(−)−orobanchol (**1**)] induced high AM fungal hyphal branching but stimulated little germination of *S. hermonthica* seeds compared with other fractions. Also, *ent*-2'*-epi-*5-deoxystrigol (**2**, fraction 28; low seed germination), the methoxy-5-deoxystrigol isomers (fractions 24–25; low seed germination) and fraction 16 and 18 (high seed germination) have very different activity with regard to the induction of seed germination whereas being quite similar in the induction of hyphal branching. Fraction 27 did not display seed germination stimulatory activity, but it did induce hyphal branching. This is probably due to the presence of *ent*-2'*-epi-*5-deoxystrigol (**2**) that is still detected in this fraction but at lower concentration than in fraction 28. Hence the concentration of *ent*-2'*-epi-*5-deoxystrigol (**2**) in fraction 27 may not be sufficient to induce *S. hermonthica* seed germination but is apparently high enough to induce AM fungal hyphal branching.

The separation on HPLC is not good enough to separate all the active compounds. This results in tailing peaks for example for (−)−orobanchol (**1**), present in fractions 19 to 21 and with highest abundance in fraction 20. Fraction 19 induces response of both *S. hermonthica* seeds and AM fungi to an extent that is intermediate to fractions 18 and 20. The activity in fraction 19 is probably a result from the cumulative effect of (−)−orobanchol (**1**) and the tail of the unknown active compound eluting mostly in fraction 18. Similarly, the activity observed in fraction 17 might also be due to fronting of fraction 18 and tailing of fraction 16. However, we cannot exclude the presence of other active compounds in fractions 17 and 19.

The two activity profiles show that some of the most active mycorrhizal hyphal branching stimulants present in rice root exudates play only a minor role in the induction of *S. hermonthica* germination. The seed germination stimulatory activity of known concentrations of SLs was assessed and compared with results of a study relating structural differences in SLs to AM fungal hyphal branching stimulatory activity [26] (Table 1). The structure of (−)−orobanchol (**1**) and (+)-*ent*-2'-*epi*-orobanchol (**6**) have been revised after the study by Akiyama et al. [26] hence, these compounds were originally labeled (+)−orobanchol and (+)-2'-*epi*-orobanchol respectively [23], [26], [31]. As previously shown by Nomura et al. [27] sensitivity of *S. hermonthica* seeds is highly dependent on the orientation of the C-ring, and is more sensitive to the strigol–type configuration. In our bioassays this preference is confirmed, the highest seed germination was obtained with sorgomol (**7**) and (+)-*ent*-2'-epi-orobanchol (**6**) while (−)−orobanchol (**1**) hardly induced any seed germination (Table 1).

AM fungi also have different sensitivity to different SL structures [26]. The strigol–type configuration is sometimes more active, as was observed for GR24 (**9a**) but not always [26]. For instance, all strigol stereoisomers (**8a**–**d**, R^1^ = CH_3_; R^2^ = OH) have equal activity just as (−)−orobanchol (**1**) and (+)-*ent*-2'-epi-orobanchol (**6**) (Table 1) [26]. Also the two natural stereoisomers of 5-deoxystrigol (**8a** and **8c**, R^1^ = CH_3_; R^2^ = H) have each the same activity at inducing hyphal branching (Table 1) [26]. Indeed, the activity of SLs to stimulate hyphal branching seems to be more influenced by modifications in rings A and B than by stereochemical variation [26], [32]. In our hyphal branching assay with a different AM species, *Gi. margarita*, we obtained a similar response to the different SLs as reported for *Gi. rosea* [26]. Fraction 20, where (−)−orobanchol (**1**) elutes, displays high activity in the branching bioassay whereas there is no clear activity peak in the seed germination bioassay (Figure 2). Similarly, *ent*-2'-*epi*-5-deoxystrigol (**2**) detected in fractions 27 and 28 induced hyphal branching and only fraction 28 with highest amounts of this SL induced low seed germination (Figure 2). SL activity is also affected by different chemical and structural properties that influence diffusion and stability [26], [33]. However compared to other SLs, (−)−orobanchol (**1**) and *ent*-2'-*epi*-5-deoxystrigol (**2**) are highly active at stimulating hyphal branching. Therefore, the low activity of (−)−orobanchol (**1**) and *ent*-2'-*epi*-5-deoxystrigol (**2**) at inducing seed germination does not seem to be a result of instability or poor diffusion of these two SLs but rather a result of lower sensitivity of the seeds to these compounds.

Our bioassays suggest that strong hyphal branching stimulators make little contribution to the overall stimulation of parasitic seed germination. Moreover, the fractions showing the largest effect on seed germination (fractions 16 and 18) contain stimulants of unknown structure. The reduction of activity in these fractions by fluridone and by mutation in *D10 (CCD8)* suggests that they are SL-like. The strong differences in activity across the exudate fractions suggest that *S. hermonthica* infection and potentially also the infection by other parasitic plant species could be reduced by altering the qualitative composition of SLs rather than just quantitatively reducing their production. New varieties with such altered SL composition could maintain their ability to establish symbiosis with AM fungi while at the same time they induce less *Striga* seed germination. In a recent study, 20 rice cultivars were screened for the abundance of SLs in their root exudates [6]. The authors observed that the relative amounts of (−)−orobanchol (**1**) and e*nt*-2'-*epi*-5-deoxystrigol (**2**) differ across cultivars, suggesting that selection for different SL composition is possible.

e*nt*-2'-*epi-*5-Deoxystrigol (**2**) and 5-deoxystrigol (**8a**, R^1^ = CH_3_; R^2^ = H) have the most simple structure of all SLs so far identified in plants. They have been suggested to be produced from carlactone through the action of MAX1 [and possibly additional enzyme(s)] [21], [34] which would imply that the orientation of the C-ring, a structural feature of major importance for the induction of seed germination as well as hyphal branching, is determined by MAX1. We propose that e*nt*-2'-*epi-*5-deoxystrigol (**2**) is the precursor for the remaining rice SLs. Further supporting this hypothesis, it has been recently shown that a sorghum enzyme(s) - likely a cytochrome P450 - converts e*nt*-2'-*epi-*5-deoxystrigol (**1**) and 5-deoxystrigol (**8a**, R^1^ = CH_3_; R^2^ = H) into e*nt*-2'-*epi*-sorgomol and sorgomol respectively [35]. Breeding for a different SL composition would be greatly aided by the characterization of these later steps in SL biosynthesis, that is the decoration of the SLs' core-structure.

As a word of caution, the sensitivity to specific SLs may vary between *Striga* species and/or races [36]. Therefore, assessment of the seed germination and hyphal branching requirements for the host/parasite and host/AM combination present in a certain region would be necessary in order to direct the development of new, locally adapted, cultivars that are less affected by *Striga* parasitism but still efficient in AM symbiosis establishment. A study performed in sorghum has shown that host plants are especially vulnerable to plant parasitism in the early stages of their life cycle [37]. Since SL composition in rice exudates changes according to the age of the plants [23] efforts to produce new rice varieties should take into consideration SL variation throughout the life cycle.

Finally, we can only speculate about the driving forces for the diversification in SL structures that we see in rice. Unlike for the gibberellins, it seems that there are not just one or two active molecules accompanied by inactive precursors and degradation products. In their role as rhizosphere signaling molecules the different SLs all display activity, albeit admittedly with different efficiency. With regard to their endogenous function, as plant hormones regulating a suit of developmental processes, we only just begin to understand the structure-activity relationships [38]. As a result of all these different functions the consequences of evolutionary and human (breeding) selection pressures are complex – which is reflected in the large structural diversification - and the resulting structural diversification so far difficult to explain.

## Supporting Information

Figure S1 **Activity profiles of rice root exudates tested with** ***S. hermonthica*** **seed germination assay.** Two biological replicates are shown here and the third replicate is shown in Figure 2 A. Crude exudates and exudate fractions from rice plants treated with full nutrition (black bars); phosphate starvation (grey bars) and phosphate starvation plus 0.01 µM fluridone (white bars). Water and SL analogue GR24 (0.005, 0.05 and 0.5 µM) were used as controls. The error bars represent the standard error of 3 technical replicates. Significance levels between treatments as determined using a *X* ^2^ test are indicated: */+  =  *P*<0.05; **/++  =  *P*<0.01; ***/+++  =  *P*<0.001; n.s.  =  *P*>0.05; *  =  control vs. phosphate starvation treatment; +  =  phosphate starvation vs. phosphate starvation plus fluridone treatment. When germination values are close to zero the statistical test cannot be performed, which is indicated with “–”.
(TIF)
