# Supplementary figures and images for: Differential Activity of Striga hermonthica Seed Germination Stimulants and Gigaspora rosea Hyphal Branching Factors in Rice and Their Contribution to Underground Communication
Source: PLoS One. 2014 Aug 15;9(8):e104201. doi: 10.1371/journal.pone.0104201 (PMC4134212; doi:10.1371/journal.pone.0104201)

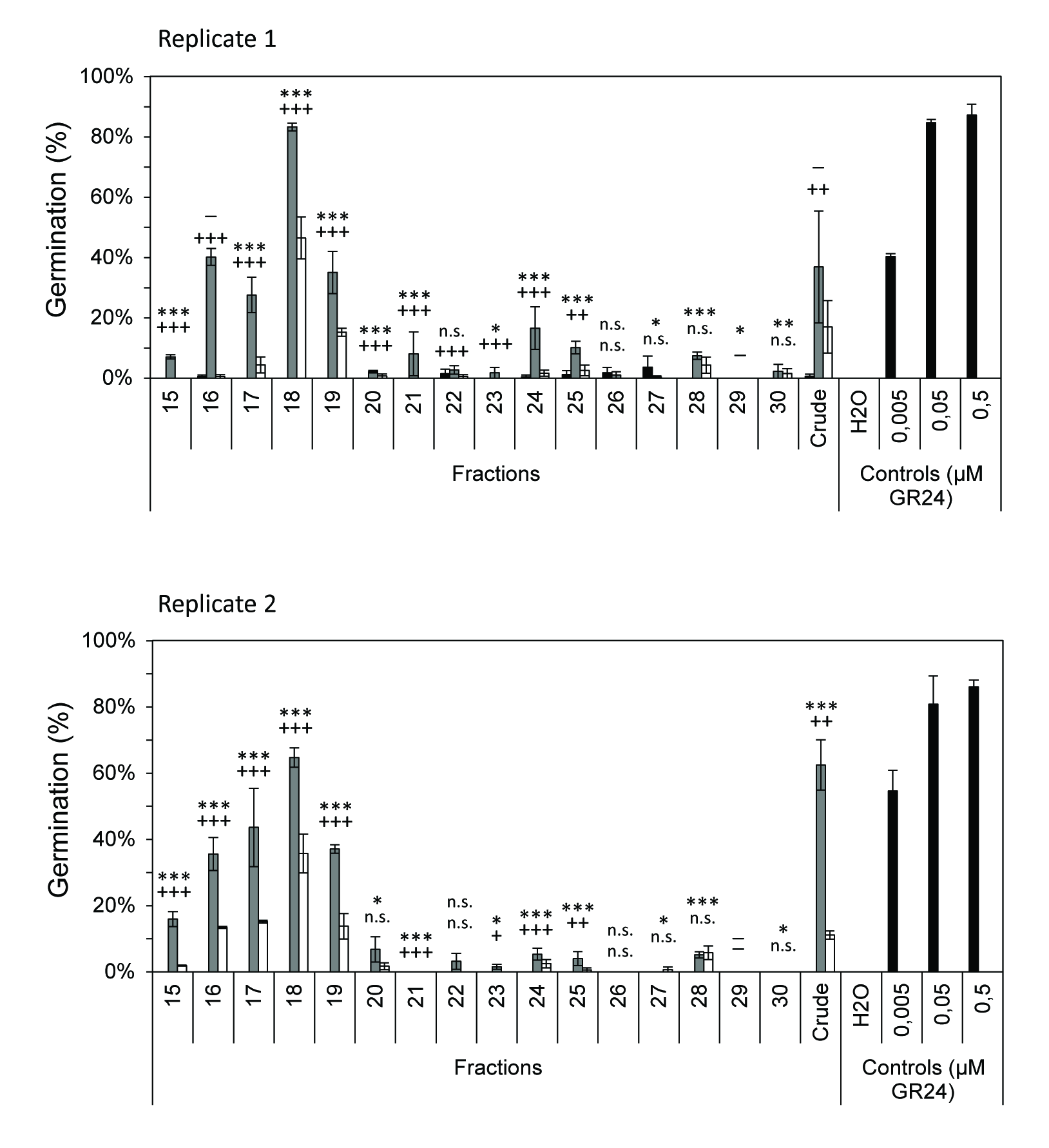

Supplement: Figure S1 — Activity profiles of rice root exudates tested with S. hermonthica seed germination assay. Two biological replicates are shown here and the third replicate is shown in Figure 2 A. Crude exudates and exudate fractions from rice plants treated with full nutrition (black bars); phosphate starvation (grey bars) and phosphate starvation plus 0.01 µM fluridone (white bars). Water and SL analogue GR24 (0.005, 0.05 and 0.5 µM) were used as controls. The error bars represent the standard error of 3 technical replicates. Significance levels between treatments as determined using a X 2 test are indicated: */+ = P<0.05; **/++ = P<0.01; ***/+++ = P<0.001; n.s. = P>0.05; * = control vs. phosphate starvation treatment; + = phosphate starvation vs. phosphate starvation plus fluridone treatment. When germination values are close to zero the statistical test cannot be performed, which is indicated with “–”. (TIF) [file pone.0104201.s001.tif]
